# Antioxidant and anti-aging role of silk sericin in D-galactose induced mice model

**DOI:** 10.1016/j.sjbs.2023.103872

**Published:** 2023-11-14

**Authors:** Shumaila Mumtaz, Shaukat Ali, Muhammad Zahid Qureshi, Ali Muhammad, Abdul Manan, Tafail Akbar Mughal

**Affiliations:** a Applied Entomology and Medical Toxicology and Laboratory, Department of Zoology, Government College University, Lahore, Pakistan; b Deanship of Educational Services, Department of Biochemistry, Qassim University, Al Qassim, Buraidah 51452, Saudi Arabia; c Department of Zoology, University of Poonch, Rawlakot, Pakistan; d Department of Zoology, Women University of Azad, Jammu Kashmir, Bagh, India

**Keywords:** Aging, Oxidative stress, Reactive oxygen species, Telomere length, Silk sericin, Antioxidant enzymes, Silkworm

## Abstract

The main purpose was to elucidate the potential anti-aging impact of sericin, due to its anti-oxidant potential in D-galactose induced mice model. To induce natural aging in mice, a solution of 0.9 % saline containing D-galactose (250 mg/kg b.w.) was injected intraperitoneally for a period of 60 days. In this experiment, 56 male mice were arbitrarily categorized into 8 groups (1: control; 2: D-Galactose (250 mg/kg b.w), Group 3: Sericin (150 mg/kg b.w), Group 4: Metformin (150 mg/kg), Group 5: sericin (P), Group 6; sericin (T), Group 7; Met (P), Group 8; Met (T). The level of Glutathione reductase (2.1 ± 0.2 µmol/L), CAT (0.5 ± 0.0 mmol/mL), Superoxide dismutase (65.4 ± 1.7 U/mL), GSHPx (69.2 ± 1.7 U/l), T3 (3.1 ± 0.7 ng/mL), IL-2 (68.8 ± 1.5 Pg/mL), IL-4 (71.4 ± 4.2 Pg/mL), IgG (0.6 ± 0.0 mg/mL) and IgM (0.6 ± 0.0 mg/mL) were significantly (P < 0.05) decreased whereas the cortisol (22.0 ± 1.5 µg/L), and total cholesterol (229.4 ± 4.2 mg/dL)) were significantly elevated in D-galactose-treated /aged mice. However, administration of sericin significantly reduced the level of oxidative stress in aged mice. Real-time qPCR data showed that the level of telomere length- gene TERT significantly downregulated (10.43 ± 0.1) in the D-Gal-treated mice with respect to control (21.97 ± 0.5). The highest significant upregulation was found in the TERT gene when D-Gal-induced aged mice were treated with sericin (24.74 ± 0.3). Our outcomes showed that sericin gradually recovered the organ indices, and improved the histological changes of the brain, kidney, and liver in D-Gal-induced aging mice. Therefore, concluded that sericin possesses anti-aging effect against D-Gal-induced aging by diminishing oxidative stress, restoring the immune system, and enhancing the antioxidant defense system.

## 1 Introduction

In multicellular organisms aging is a degenerative, time-dependent universally conserved biological process, characterized by various risk factors (neurodegenerative diseases, cardiovascuLar diseases, cancers, metabolic syndrome, poor immune system, and directly affect skin appearance) that increase the morbidity and mortality rate. Oxidative stress due to excess formation of free radicals or ROS is the most significant determinant of aging and these ROS are attributed to oncogene activation, increase oxidation of biomolecules, telomere shortening, decreased in the antioxidant defense system (SOD, CAT, GSHpx, GSHRx), and the function of mitochondria and DNA damage (Mumtaz et al., 2021). D-galactose is a reducing carbohydrate that equitably exists in the physique and at normal concentrations, it undergoes complete metabolism (Alejandro, 2022). However, at higher concentrations in the presence of galactose oxidase and aldose reductase, it is transformed to hydrogen peroxide, D-galactose-hexodialdose, hydrogen peroxide, and galactitol respectively, hence increasing the production of free radicals e.g., superoxide anion etc (Guo et al., 2020). When free radicals accumulate, they can cause oxidative stress by promoting the aging process through increased lipid oxidation, proteins, and DNA (Luo et al., 2023). Since 1985, researchers have utilized human fetal lung fibroblasts, rats, mice, and *Musca domestica* as models to study aging (Men et al., 2021).

It has also been identified that the introduction of D-galactose in mice leads to accelerated aging and increased production of oxidants, accumulation of oxidative damage, changes in antioxidant enzyme activity as well as inflammation, tissue injury, cognitive impairment, increased ROS production, neurotoxicity, and reproductive aging (Chelliah et al., 2021). Maintaining a healthy biological system requires a balance between free radicals and redox equilibrium. Antioxidants are synthetic or natural molecules that can quench the free radicals found in the physique at very low concentrations (Lee et al., 2021). According to Falowo et al. (2014), the endogenous antioxidant system, which includes superoxide dismutase, hydrogen peroxide, and catalase, can prevent the harmful effects of reactive species. In living organisms, antioxidant compounds play a role in suppressing the oxidant molecules. Many plants and other species produce these substances as a result of their metabolic processes, and the human body can also receive them by taking supplements from outside sources (Sevindik et al., 2017, Selamoglu, 2018). Studies have shown that the antioxidant properties of plant extracts might be due to the quantitative and qualitative compositions of the nutrients and phenolic components. Furthermore, the pharmaceutical industry frequently uses natural foods and materials because they are thought of as a fundamental source of medications. Due to this condition, there is a greater need than ever for medicinal plants in the field of natural medicine. Therefore it is a global demand that new plants and food sources are being explored and exploited for their potential health and nutritional benefits (Sevindik et al., 2017, Michalak et al., 2021).

Sericin has been demonstrated to act as a scavenger by stabilizing the free radicals that cause oxidative damage (Thomas et al., 2021). Kim and colleagues (2021) found that sericin, which is derived from Bombyx mori cocoon, has the ability to scavenge around 80 % of reactive oxygen species (ROS). Sericin was also observed to have a mitigating effect on oxidative stress induced by alcohol consumption in the livers of mice (Li et al., 2019). In addition, Thomas et al. (2021) have reported that sericin exhibits higher antioxidant activity compared to vitamins C and E, which are known for their antioxidant properties. Miguel and Álvarez-López (2020) reported that sericin has important biological features that allow it to be used in a wide range of applications. Several publications have documented its prevention of oxidation of lipids, anti-tyrosinase action, and nuLlification of excess formation of ROS (Miguel and Álvarez-López, 2020). Sericin protein's antioxidant activity varies depending on the extraction process employed. Sericin has been found to improve constipation in mice, enhance mineral uptake inside intestines, and also have a prebiotic effect (Sharma et al., 2021). Dong et al. (2020) investigated the hypoglycemic activity of sericin protein in type 2 diabetic (T2D) rats by orally administering it at a concentration of 0.8 percent along with a regular meal. The silk sericin substantially reduced fasting sugar levels, fasting blood insulin levels, and concentrations of glycosylated serum; enhanced oral insulin and glucose tolerance, and increased antioxidant potential. The protein may help to repair pathological deterioration within cells of the pancreas and liver (Rahbarghazi et al., 2021). Generally, sericin may be able to sustain appropriate glucose levels while also regulating insulin production, insulin metabolism, and prevention of inflammation (Dong et al., 2020). The purpose of this research was to create innovative treatments for combating the effects of aging. In this study, silk sericin was utilized as a unique anti-aging substance to counteract the aging-induced in a mouse model by D-Galactose.

## 2 Research methodology

### 2.1 Degumming of sericin

**Supplementary Fig. 1:**
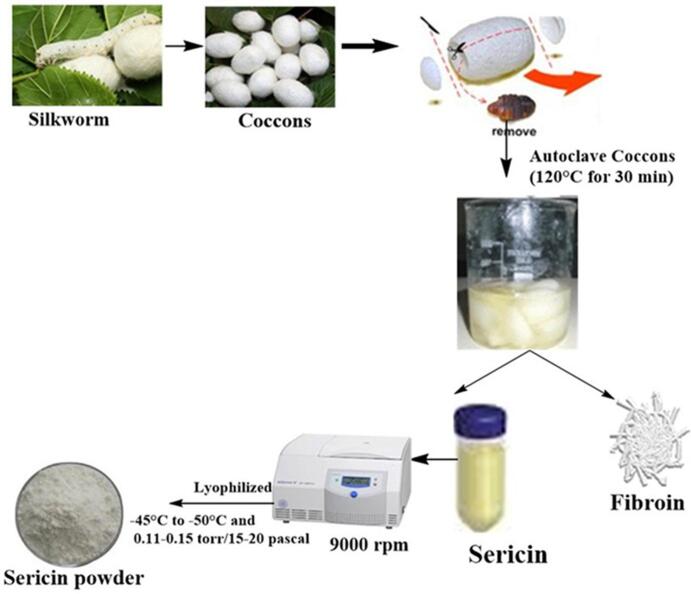
Extraction of silk sericin

Supplementary Fig. 1 illustrates the utilization of the autoclave method for extracting silk sericin from the cocoons of *Bombyx mori* (silkworms) as described in our latest publication (Mumtaz et al., 2023).

### 2.2 Antioxidant activities

The ability of sericin to scavenge the 2,2-Diphenyl-1-picrylhydrazyl (DPPH) radical.

Free radical scavenger activity of sericin was assessed at numerous concentrations (0.5 mg, 1 mg, 2 mg/mL), and vitamin C was used as a standard as described by (Mumtaz et al., 2023).

#### 2.2.1 The silk sericin's overall phenolic content

A modified version of the technique developed by Napavichayanun et al. (2017) was employed to enumerate the total phenolic content of silk sericin. This modified method utilizes Folin-Ciocalteu chemical and gallic acid as a standard for the quantification process. Briefly, 100 μLof various concentrations (0.5–2 mg/mL) of sericin was first thoroughly mixed with 100 μLdouble distilled water in falcon tubes. Next, the Folin-Ciocalteu reagent (1 mL) was gently mixed in. After a 5-minute interval, 1 mL of (4 %) Na2CO3 was added and the volume was adjusted with deionized water to reach the desired level. Solutions were shaken and kept at 40 °C for 90 min. All gallic acid standard solutions were repeated with the same method. A spectrophotometer (Perkin Elmer Lambda 35 ESUV/Vis) was used to measure absorbance at 760 nm. All samples were analysed in triplicates and the graph was plotted concentration vs absorbance.

#### 2.2.2 Ferric ion reducing antioxidant power assay or FRAP assay

To prepare the fresh stock solution of the FRAP reagent, a precise chemical process was undertaken. The procedure required combining 100 mL of acetate buffer with a concentration of 300 mM and a pH of 3.6, along with 10 mL of a solution containing 40 mM of 2, 4, 6-tripyridyltriazine (TPTZ) dissolved in HCl, and 10 mL of a strong 20 mmol FeCl3 solution, using a specific ratio of 10:1:1.The resulting mixture was then gently warmed at a temperature of 37 °C for a 1 h. Numerous concentrations of sericin were prepared in deionized water and 150 μLof sericin solution of different concentrations was separately added to 3 mL of FRAP reagent. Solutions were homogenized by shaking and kept at 58 25 °C for half an hour. Then OD was calculated at 590 nm against a control. Ferrous Sulfate (FeSO4) was used as a standard to estimate the reducing activity of sericin to convert Fe3 + to Fe2+ (Sangwong et al., 2016).

#### 2.2.3 Measurement of the overall flavonoid content

The total flavonoid content in silk sericin was assessed by a previously reported method with minor modifications (Anuduang et al., 2020). Different concentrations of silk sericin were prepared (0.5, 1, and 2 mg/mL) in deionized water. Double distilled water 130 μLwas added in 25 μLof each concentration of sericin followed by the addition of 10 μLof 4 % sodium nitrite. The solutions were allowed to remain at room temperature for a duration of 10 min. After incubation, a mixture of 20 μLof 7 % AlCl3 was placed at room temperature for 8 min. The reaction was stopped by the addition of sodium hydroxide (60 μL) of 1 mM. At 510 nm absorbance was determined by using Spectrophotometer. Catechin was used as a standard and the same method was used to prepare the dilutions of catechin. A standard solution was prepared in the absence of an antidote (sericin).

### 2.3 Animals and their maintenance

We acquired male Swiss albino mice that were eight weeks old and weighed 32 g from the Animal House Facility at Government College University Lahore, Department of Zoology. Ethical guidelines of GC University Lahore were followed for the care and handling procedures. Mice were given unrestricted access to food and water in a controlled setting and were enabled to acclimatize in the laboratory for two weeks before the beginning of the experiment.

### 2.4 Ethical clearance

This experimental animal study was conducted after obtaining ethical consent, which was approved under letter No. (GCU-IIB-1003/2019).

### 2.5 Measurement of body mass

At the commencement of the investigation, the mice's bodily mass was meticulously recorded using an electrical weighing apparatus on an individual basis. Subsequently, throughout the duration of the experiment, the body weight of both the control and treatment groups was assessed on a weekly basis. The mass of every animal was subsequently measured and recorded in grams. The weight of each animal was then quantified in grams (g). To ensure accurate dosing, the mice were administered doses under their respective average body weights. As the experiment progressed, fresh doses were prepared following the newly updated average body weights of the mice.

### 2.6 Induction of aging in mice model

**Fig. 1:**
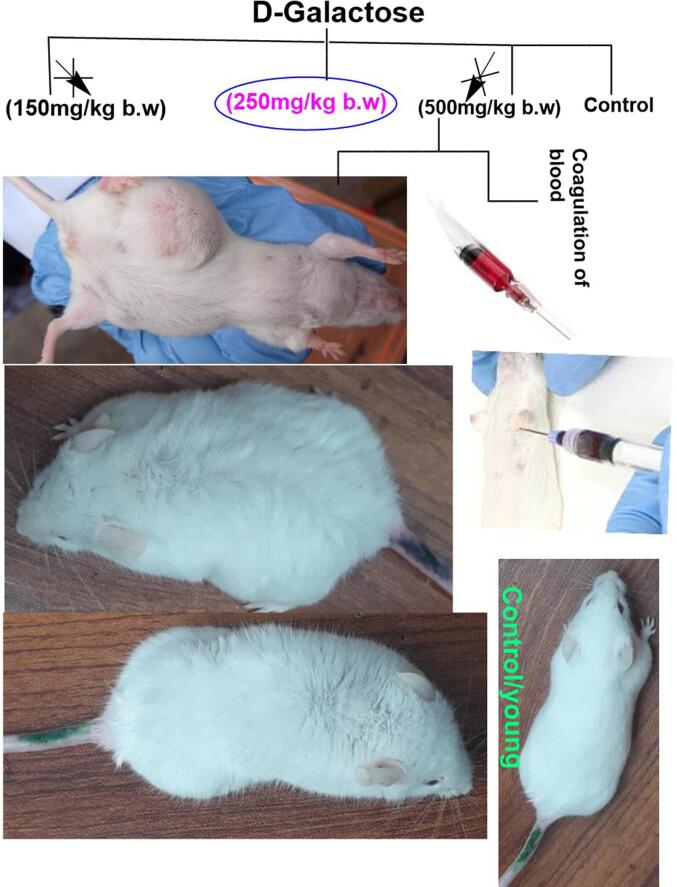
Selection of doses based on trial experiment.

D-galactose (250 mg/kg body weight) was dissolved in 0.9 percent normal saline and injected 0.3 mL intraperitoneally for 60 consecutive days to produce a natural aging mice model (Garcez et al., 2021). For induction of aging or in anti-aging pharmacological research route of exposure and daily dose have been well known (Hassani et al., 2020). The dose of D-Galactose (250 mg/kg b.w.) was selected based on the literature review and conducting a trial experiment for 60 days. At (500 mg/kg b.w.) clot formation was found during blood sampling so this dose was excluded as shown in Fig. 1. Doses of sericin were selected according to 5 % LD50 of sericin which was 150 mg/kg b.w.), and metformin 150 mg/kg b.w on literature-based (Novelle et al., 2016, Johnson et al., 2020).

### 2.7 Grouping of animals

**Fig. 2:**
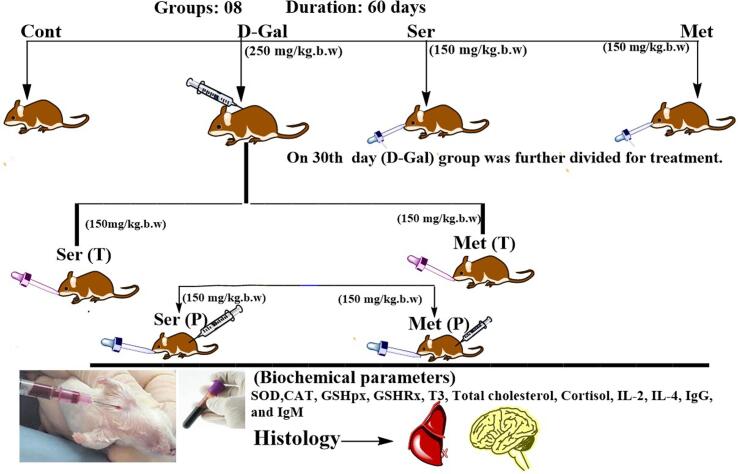
Diagrammatic representation of study.

Experimental design and grouping of animals In the present study, 60 days were selected for the experimental duration to induce aging and for further treatments. After 2 weeks of adjustment, 48 mice were arbitrarily categorized into 08 groups (One control group and 07 treatment groups). Each group contained six replicates. They were categorized according to body weight and treated as follows as shown in Fig. 2. Group 1 consists of six (06) animals and this group received 0.3 mL of normal saline solution for a duration of eight weeks. In Group 2, the animals were orally administered with 0.3 mL of sericin (at a dosage of 150 mg/kg b.w) throughout the entire experiment (Yellamma, 2014). As for Group 3: Metformin was administered to the mice orally at a dosage of (150 mg/kg b.w) as a positive standard drug for 60 days. Group 4: Mice were treated with an intraperitoneal injection of 0.3 mL of (250 mg/kg b.w) D-Galactose for 60 days or till the end of the experiment. Groups 5–6: (Ser (T), and Met (T) are post-treatment groups. For the first 30 days, these post-treatment groups were given intraperitoneal injections of D-Gal at a dose of 250 mg/kg body weight once daily each day, and from the 31st day mice were treated with (150 mg/kg b.w) orally administration of (sericin, and (150 mg/kg b.w) of metformin, respectively for 30 days or up to end of the experiment. Groups 7–8: Ser (P), and Met (P) are Pre-treatment or prevention groups. These groups were treated at the same time with D-Gal (250 mg/kg b.w; I.P) and oral administration of (sericin and metformin) respectively for 60 days or up to the end of the experiment (Yellamma, 2014, Seyedaghamiri et al., 2021).

### 2.8 Blood sampling

Following a period of 60 days, the animals were sacrificed and blood samples were obtained by performing a cardiac puncture following a span of following a 24-hour fasting period. For serum isolation blood samples were spun at 4 °C and 2000 rpm for 15 min. Blood serum was preserved at −20 °C for further study of biomarkers linked to stress and aging (Umbayev et al., 2020).

### 2.9 Extraction of DNA

DNA from the whole blood of mice was extracted by using a solution composed of phenol, chloroform, and isoamyl alcohol in a ratio of 25:24:1. This was done through a qPCR procedure, as described by Kahl et al. in 2020. (Kahl et al., 2020). Fresh blood was collected in EDTA tubes and then diluted in 1:1 with TE buffer (pH 7–8) and slightly overturned to mix. The samples were centrifuged at a speed of 12,000 rpm for a duration of 10 min and the pellet was resuspended in buffer and centrifugation process was repeated until its color became pinkish white. Afterward, the pellet underwent treatment with sodium acetate, SDS, and proteinase K, which was then thoroughly mixed by inverting. Then kept overnight for incubation at 37 °C. Afterward treated with phenol/chloroform/isoamyl alcohol (25:24:1) solution for the separation of a layer. Then centrifuged at 12000 rpm for 10 min. The lymphocyte layer at the interface containing DNA was transferred into a fresh tube using a pipette. Then isopropanol was added for precipitation of DNA and kept for one hour at room temperature. Then centrifuged at a speed of 12000 rpm for 10 min and the supernatant was discarded and the pellet encompassing DNA was resuspended in ethanol and incubated at room temperature for 10 min. Centrifugation was repeated and the pellet was dried and extracted DNA was re-suspended in TE buffer for long-term storage at −80 °C. Nanodrop was used to quantify DNA samples by using the 260/280 ratio (1.7–1.8). Agarose gel electrophoresis was used to assess the DNA quality (Joglekar et al., 2020).

### 2.10 qPCR for measurement of telomere length

Real-time PCR quantitative analysis (qPCR) was used to measure the average telomere length of two genes (beta-actin/Actb and Telomerase reverse transcriptase /TERT) by using the DNA of mice. Before performing qPCR, each DNA sample and primers were diluted in nuclease-free water to the required concentration. To reduce inter-assay variability single-copy gene/β-actin and the telomere TERT were analyzed on the same plate. The PCR reaction mixture (15 µL) consists of 5 µL of 100 ng/30 µL of DNA, forward and reverse primers; 2 µL, Syber Green; 7.5 µL, and 0.5 µL of nuclease-free water (Wang et al., 2018; Joglekar et al., 2020).

The primer sequences for Actb and TERT were: TERT-F 5′-GGATTGCCACTGGCTCCG-3′ TERT-R 5′-TGCCTGACCTCCTCTTGTGAC-3′ (Muñoz-Lorente et al., 2019). Actb-F: 5′-GCTCTTTTCCAGCCTTCCTT-3′ Actb-F: 5′-CGGATGTCAACGTCACACTT-3′ (Wang et al., 2018b). The PCR SYBR green master mix was mixed vigorously and then centrifuged prior to being added to the PCR plate. Two master-mixes were prepared one for housekeeping gene Actb primers and the other for TERT primers. Prepared PCR mixture 15 µL was added into each well. All samples were run in duplicate and one reference DNA sample/ control DNA was used in each plate. Then the plate was sealed via optical adhesive film to minimize the generation of any aerosols (Joglekar et al., 2020). At 4 °C the plate was spun in a centrifuge at a rate of 3000 revolutions per minute for a duration of 5 min to eliminate the air bubbles. In the Real-Time PCR system, the plate was inserted and PCR software on the computer loaded up. The correct sample and target names were entered in wells. In the Actb and TERT PCR experiments, an annealing temperature of 60 °C was employed. The Light Cycler 480 instrument by Roche Diagnostics GmbH was used to carry out the amplification reactions. The reactions began with an initial hold at 95 °C for 10 min, followed by 40 cycles of a three-step PCR process: 95 °C for 15 s, 60 °C for 15 s, and 72 °C for 30 s. To ensure there were no primer dimers present, melt curve analysis was conducted after 40 cycles of PCR. The measurement of relative telomere length involved calcuLating the threshold (Ct) values for Actb and telomere genes and then results were analyzed by GraphPad Prism and Excel (Scarabino et al., 2019, Cheng et al., 2020).

### 2.11 Determination of catalase (CAT) levels

Tkachenko et al. technique was used to measure the activity of catalase spectrophotometrically. The sample (10 μL) was mixed with 100 mol/mL of hydrogen peroxide in Tris-HCl buffer (0.05 mmol/l, pH 0.7) for 10 min. Then 50 μL of 4 % ammonium molybdate was mixed and a yellow complex containing H_2_O_2_ and ammonium molybdate was detected at 410 nm (Tkachenko et al., 2019).

### 2.12 Assay for glutathione reductase levels

The activity of glutathione reductase was measured at 30 °C by the rate of oxidation of NADPH as designated by (Renó et al., 2021). The mixture of reaction consists of a required sample, a 100 mM potassium phosphate buffer with a pH of 7.5, 0.5 mM EDTA, and 0.1 mM NADPH.

### 2.13 Determination of glutathione peroxidase (GSH Px) levels

The activity of glutathione peroxidase was dignified following the oxidation of NADPH and in the presence of GR and GSH at room temperature. Sample (10 μL) of was added to the reaction mixtures (2 mM GSH, 1 mM sodium azide, 0.2 mM NADPH, 50 mM potassium phosphate, 1.5 mM cumene hydroperoxide, and 1 U/mL GR). After that at 25 °C samples were incubated for 5 min and absorbance was calculated at 340 nm (Iske et al., 2018).

### 2.14 Determination of superoxide dismutase (SOD) levels

The hemolysates were used to determine the activity of SOD in the occurrence of xanthine oxidase and absorption was calculated at 505 nm as reported by (Tkachenko et al., 2019).

### 2.15 Determination of total cholesterol level

A commercial cholesterol kit was used for the estimation of total cholesterol (TC) in serum following the method of Mohamed. The serum sample (10 µL) was mixed with Cholesterol oxidase Peroxidase, Phenol derivate, and Cholesterol esterase and kept for 10 min at 37 °C and optical density was calculated at 546 nm (Mohamed et al., 2018).

### 2.16 Measurement of cortisol and T3

The levels of serum cortisol were measured according to the manufacturer’s guidelines (SenbeiJia Biological Technology Co., Ltd, Nanjing, China) by using ELISA Kits (Zhang et al., 2021). ELISA kit was used to measure the T3 level in serum according to the instructions of Xiamen Huijia Bioengineering Institute, Xiamen, China (Chu et al., 2019).

### 2.17 Serum IL-2, IL-4, IgG and IgM

The serum contents of IgG, IgM, IL-2, and IL-4, were analyzed by ELISA kits (Li et al., 2020).

### 2.18 Histological analysis

During the dissection process, the kidneys, liver, and brain were removed surgically and to ensure cleanliness, they were then washed with a solution called phosphate buffer saline, which helped to remove any debris. Each organ was then weighed in grams using a highly accurate analytical balance. Following the weighing process, these organs were placed in a fresh solution containing 10 % formalin for a period of 24 h to undergo fixation, which had a pH level of 7.4. Next, a dehydration process was carried out by gradually increasing the concentration of alcohol (70 % alcohol, then 90 %, and finally 100 %). Once the alcohol had fully dehydrated the organs, it was cleared using a substance called xylene. Then organs were fixed in paraffin wax, which was heated to a temperature of 60 °C. Blocks were created, and sections measuring 5 mm in thickness were sliced using a microtome apparatus. To enhance visibility and highlight specific features, a staining process of hematoxylin and eosin (H&E) was carried out on all of the tissue sections. Once the sections were properly stained, they were examined under a light microscope (Feng et al., 2020).

### 2.19 Statistical analysis

The means ± SEM were used to calculate the results. The numerical calculation was accomplished by GraphPad Prism (version 5 and using SPSS (version 23). The data was evaluated using the Tukey multiple comparison test and one-way analysis of variance (ANOVA) with the Bonferroni test. If the P-value was less than 0.05, the values were considered to be statistically substantial.

## 3 Results

### 3.1 Antioxidant activity of silk sericin

**Fig. 3:**
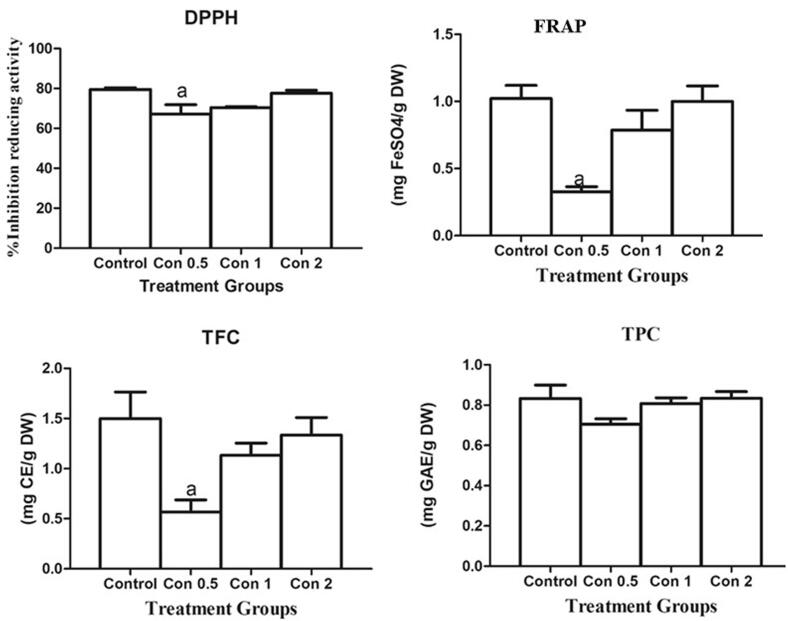
**Analysis of DPPH: 1,1-diphenyl-2-picrylhydrazyl free radical scavenging activity.** FRAP: Ferric reducing antioxidant activity, TFC: Total flavonoid content, and TPC: Total phenolic content. Abbreviation and keys: Con represent “concentration”, “a” represents the significant difference between con 0.5 and control group.

The free radical foraging action of the silk sericin and ascorbic acid is quantified through % inhibition as described in our latest article (Mumtaz et al., 2023). At a concentration of 2 mg/mL, silk sericin exhibited the greatest inhibition activity (73.33 ± 4.41 %), while no noteworthy variance in inhibition activity was detected among silk sericin and the control group (80.67 ± 2.3 %) as depicted in Fig. 3.

#### 3.1.1 Ferric reducing antioxidant effect of sericin

The ability of natural antioxidants (silk sericin) to reduce Fe^c+^ to Fe^2+^ was investigated by the use of the FRAP assay. Maximum conversion of Fe^c+^ to Fe^2+^ was shown by silk sericin (0.69 ± 0.25 mg FeSO_4_/g DW) at the highest concentration (2 mg/mL), which is a symptom of better-reducing action. A non-significant difference was found in the ferric-reducing ability of silk sericin with respect to the control group as shown in Fig. 3.

#### 3.1.2 Total flavonoid content

**Fig. 4:**
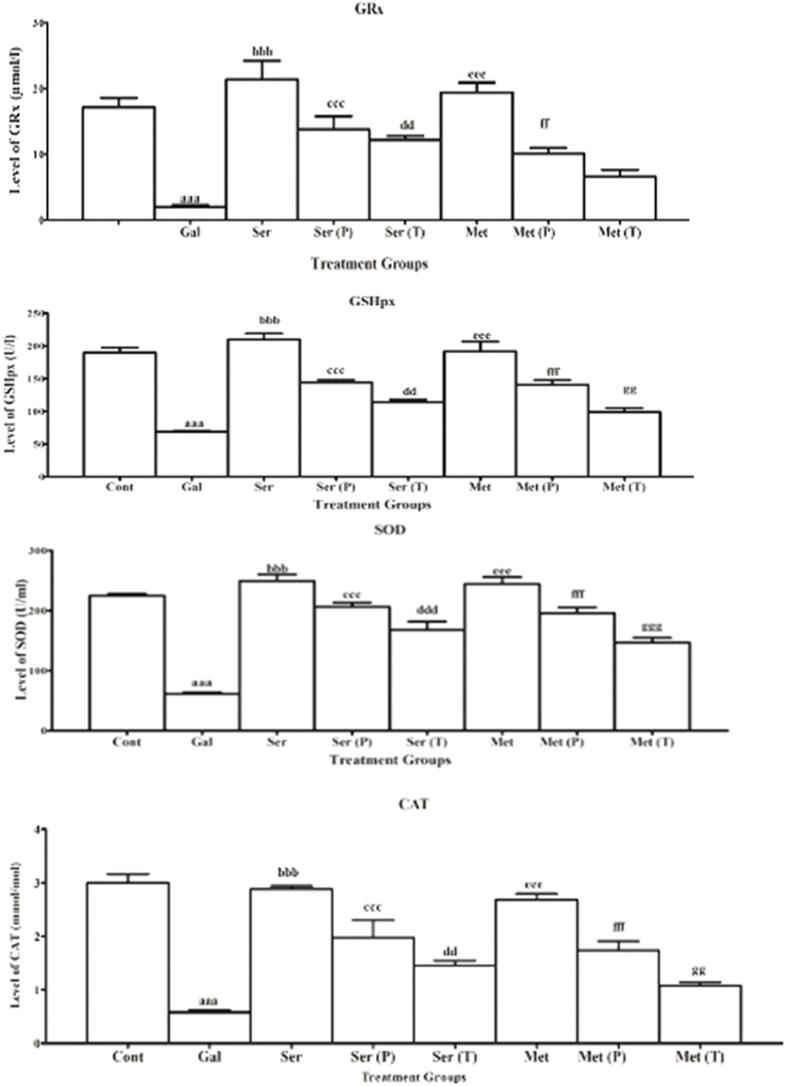
**Effect of silk sericin on antioxidant enzymes**: Glutathione Reductase (GRx), Glutathione peroxidase (GSHpx), Superoxide dismutase (SOD), Catalase (CAT). **Abbreviations and keys:** ‘**Cont**’ for control, ‘**D-Ga**l’ signifies the D-galactose, ‘**Met**’ for metformin, **‘P**’ for prevention, ‘**T**’ for treatment. “a” depicts the difference between **Cont** and **D-Ga**l. ‘b’ designates the noteworthy variance among D-Gal and ser, ‘c’ indicates the substantial variance among **D-Gal** and **ser (P)**, ‘d’ exhibits the substantial variance among **D-Gal** and **ser (T)**, ‘e’ indicates the significant difference between **D-Gal** and **Met,** ‘f’ directs the noteworthy variance among **D-Gal** and **Met (P).** ‘g’ directs the noteworthy variance among **D-Gal** and **Met (T). Numerical signs: a,b,c,d,e,f,g (p ≤ 0.05)**; aa,bb,cc,dd,ee,ff,gg **(p ≤ 0.01)**; aaa, bbb, ccc, ddd,eee,fff,ggg **(p ≤ 0.001).**

Total flavonoid content in the silk sericin in comparison to a positive control (Catechin) are shown in Fig. 4.Total flavonoid content in silk protein was measured at various concentrations (0.5 mg/mL, 1 mg/mL, and 2 mg/mL). The concentration of silk sericin was found to have a positive correlation with the TFC (total flavonoid content). Silk sericin showed TFC at each concentration but higher flavonoid contents of silk sericin 1.33 ± 0.18 mg CE/g DW were found at the highest concentration 2 mg/mL with respect to other concentrations (0.5 and 1 mg/mL). Substantial variance in flavonoid contents of silk proteins was not found with respect to control group.

#### 3.1.3 Overall phenolic content of sericin

The total phenolic content in silk sericin at different concentrations (0.5, 1, and 2 mg/mL) was measured using the Folin-Ciocalteu chemical with gallic acid as a standard. A negligible variation in the total polyphenol content (TPC) of silk sericin was observed when comparing it to the control group. However, when the concentration of silk sericin increased from 0.5 to 2 mg /mL the values of TPC increased from (0.71 ± 0.03 mg GAE/g DW) to 0.81 ± 0.03 mg GAE/g DW respectively. The TPC contents were found in silk sericin equal to positive control as shown in Fig. 3.

### 3.2 Effect on GSHRx

In the present study, the introduction of 250 mg/kg body weight of D-galactose through intraperitoneal injection for 60 days caused in a notable diminution in the GSHRx level (2.1 ± 0.2 µmol/L) in contrast to the control group (18.0 ± 1.1 µmol/L). The level of GSHRx was found highest (21.4 ± 2.9 µmol/L) when mice were treated with individual sericin orally, as shown in Fig. 4. However, Co-administration of sericin, and metformin with D-Gal improved the level of GSHRx (Ser (P): 13.8 ± 2.0 µmol/L Met (P): 10.0 ± 0.8 µmol/l) when compared to aged/ D-Gal group (2.1 ± 0.2 µmol/L). Non-significant difference (6.4 ± 1.1 µmol/L) was found in the post-treatment group of Met (T) in comparison to the D-Gal group. The outcomes specified that the GSHRx levels in the pretreatment groups were nearly equivalent to those of the normal untreated group. In contrast, the post-treatment groups exhibited a substantial reduction in GSHRx levels in the serum of the mice, particularly in the group treated with sericin at a prescription of 150 mg/kg b.wt, where it reached a level of 12.2 ± 0.6 µmol/L.

### 3.3 Effect on SOD

The administration of D-galactose (250 mg/kg b.w.) intraperitoneally for 60 sequential days result in the most significant decrease (65.4 ± 1.7 U/mL) in SOD levels when compared with a control group (225.2 ± 3.5 U/mL). A noteworthy upsurge in the level of SOD was detected in the serum of mice that received oral treatment of 150 mg/kg body weight of Ser: 249.2 ± 10.9 U/mL and Met: 242.2 ± 6.3 U/mL as compared to D-Gal group (65.4 ± 1.7 U/mL). Highest significant elevation in SOD levels (Ser (P): 206.2 ± 7.2 U/mL; Met (P): 194.6 ± 9.8 U/mL was perceived in the prevention/Pre-treatment groups when compared with D-Gal group (65.4 ± 1.7 U/mL). However, the SOD in the serum of mice expressively declined to the highest extent in post-treatment groups, but this restoration was less than in all pre-treatment and individual groups as illustrated in Fig. 4. Maximum significant elevation in the level of SOD was found in a post-teratment group of (Ser (T): 174.8 ± 8.4 U/mL; Met (T): 145.2 ± 8.5 U/mL with respect to D-Gal group (65.4 ± 1.7 U/mL).

### 3.4 Effect on GSHPx

In this study, it was found that administering D-galactose at a dosage of 250 mg/kg body weight through intraperitoneal injection for a duration of 60 days revealed a substantial decline in the level of GSHPx (69.2 ± 1.7 U/l) in comparison to the control group (189.4 ± 7.7 U/l). The level of GSHPx showed the highest significant increase (210.0 ± 9.2 U/l) when mice were treated orally with individual sericin, as depicted in Fig. 4. However, when sericin and metformin were administered together with D-Gal, the level of GSHPx increased (Ser (P): 144.4 ± 3.8 U/l and Met (P): 140.6 ± 7.4 U/l) compared to the D-Gal group (69.2 ± 1.7 U/l). Non-significant difference (99.0 ± 6.2 U/I) was found in the post-treatment group of Met (T) in comparison to the D-Gal group (69.2 ± 1.7 U/l).

### 3.5 Effect on catalase

Administration of D-galactose (250 mg/kg body weight) via intraperitoneal caused the highest significant decline (0.5 ± 0.0 mmol/mL) in catalase levels when compared with a control group (3.3 ± 0.2 mmol/mL). The highest substantial elevation in catalase level (Ser: 3.2 ± 0.1 mmol/mL; and Met: (2.7 ± 0.1 mmol/mL) was found with respect to D-Gal group (0.5 ± 0.0 mmol/mL). The highest significant elevation (2.5 ± 0.2 mmol/mL) in catalase level was observed in Ser (P) and Met (P) (1.7 ± 0.2 mmol/mL) when compared with the D-Gal group (0.5 ± 0.0 mmol/mL). Whereas, in post-treatment groups of sericin, the level of catalase was found to be enhanced but this elevation was lower than in prevention or pre-treatment groups. The level of catalase in the Met (T) group showed no significant difference, with an average measurement of (1.1 ± 0.0 mmol/mL) with respect to D-Gal group as revealed in Fig. 4.

### 3.6 Effect on cortisol

**Fig. 5:**
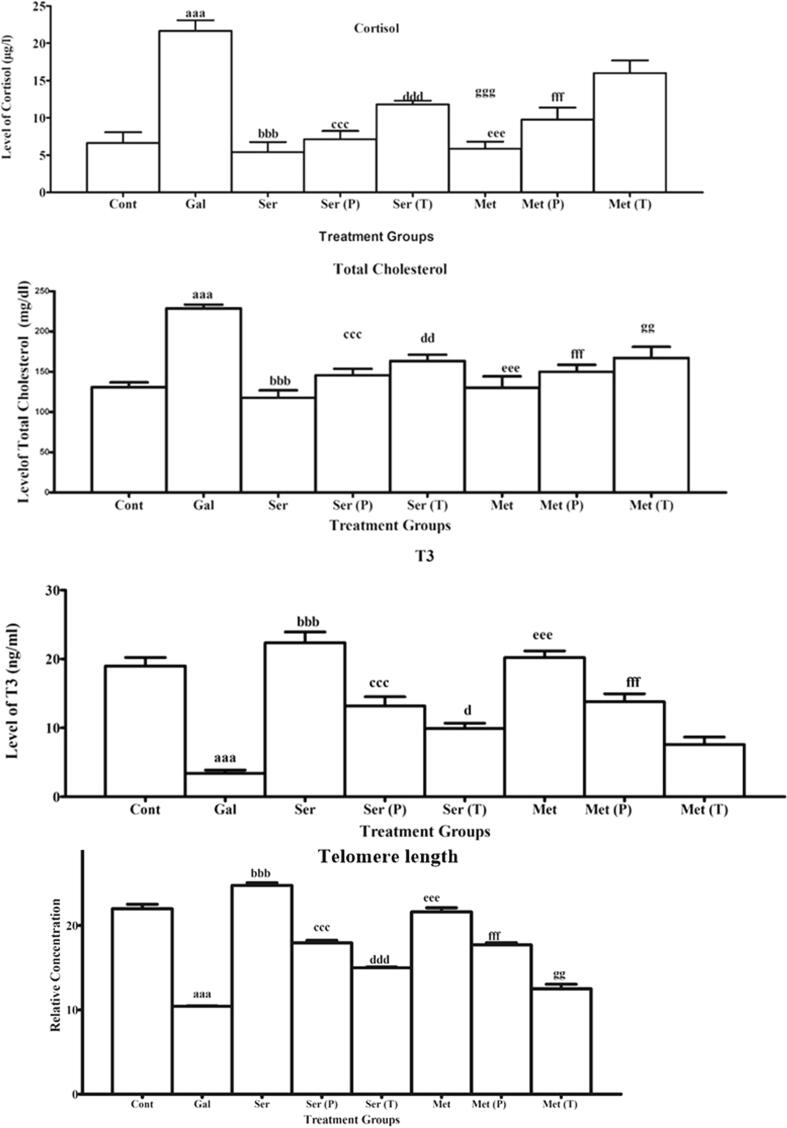
**Effect of silk sericin on cortisol, total cholesterol, Tri-i0dothyronine (T3) and Telomer length (TERT). Abbreviations and keys:** ‘**Cont**’ for control, ‘**D-Ga**l’ signifies the D-galactose, ‘**Met**’ for metformin, **‘P**’ for prevention, ‘**T**’ for treatment. “a” depicts the difference between **Cont** and **D-Ga**l. ‘b’ designates the noteworthy variance among D-Gal and ser, ‘c’ indicates the substantial variance among **D-Gal** and **ser (P)**, ‘d’ exhibits the substantial variance among **D-Gal** and **ser (T)**, ‘e’ indicates the significant difference between **D-Gal** and **Met,** ‘f’ directs the noteworthy variance among **D-Gal** and **Met (P).** ‘g’ directs the noteworthy variance among **D-Gal** and **Met (T). Numerical signs: a,b,c,d,e,f,g (p ≤ 0.05)**; aa,bb,cc,dd,ee,ff,gg **(p ≤ 0.01)**; aaa, bbb, ccc, ddd,eee,fff,ggg **(p ≤ 0.001).**

During a period of 60 days, D-galactose was administered through intraperitoneal injection at a dosage of 250 mg/kg body weight, and the highest significant increase (22.0 ± 1.5 µg/L) was found in the level of cortisol in mice treated with D-Gal with respect to group that was untreated (6.5 ± 1.4 µg/L) The highest noteworthy reduction in the level of cortisol was observed as follows (Ser: 5.4 ± 1.4 µg/L; and Met:6.5 ± 0.8 µg/L) in comparison to aged/ D-Gal treated mice (22.0 ± 1.5 µg/L). On the other hand, in pre-treatment groups sericin (6.9 ± 1.1 µg/L) and metformin (10.5 ± 1.3 µg/L) showed the highest significant decline in the level of cortisol in relative to aged mice (22.0 ± 1.5 µg/L). However, in the post-treatment group of sericin (Ser (T): 11.8 ± 0.5 µg/L) highest significant attenuation was found in the cortisol level of mice but less than in pre-treatment groups as presented in Fig. 5.

### 3.7 Effect on total cholesterol

Supplementation of D-galactose (250 mg/kg body weight) via intraperitoneal for 60 days caused a noteworthy upsurge (229.4 ± 4.2 mg/dL) in total cholesterol level when matched to the control group (131.2 ± 6.0 mg/dL). However, when sericin was given orally (150 mg/kg body weight) to mice, the highest significant decline (117.8 ± 9.2 mg/dL) was recorded as compared to D-Gal group (229.4 ± 4.2 mg/dL). Administration of metformin also decreased the level of total cholesterol but is lower than sericin. On the other hand, administration of sericin along with D-Gal the highest substantial reduction in total cholesterol was observed in Ser (P): (145.6 ± 8.2 mg/dL) when compared to D-Gal group (229.4 ± 4.2 mg/dL). However, the highest significant attenuation was noticed in the level of total cholesterol in the post-treatment group of Ser (T): (163.4 ± 7.8 mg/dL) as compared to D-Gal induce aging group (229.4 ± 4.2 mg/dL) as revealed in Fig. 5.

### 3.8 Effect on T3

In the present study, administration of D-galactose (250 mg/kg body weight) through intraperitoneal injection for 60 days revealed a substantial reduction in T3 in serum (3.1 ± 0.7 ng/mL) in comparison to the control group (18.6 ± 1.3 ng/mL) as illustrated in Fig. 5. When mice were treated orally with sericin individually, the level of T3 was found to be high. However, when sericin and metformin were administered together with D-Gal, either as pre-treatment or co-administration, the level of T3 increased significantly. Specifically, Ser (P) exhibited a level of (13.2 ± 1.3 ng/mL) and Met (P) exhibited a (13.8 ± 1.2 ng/mL) of level in serum compared to the D-Gal group which had a level of (3.1 ± 0.7 ng/mL). The findings indicated that the T3 levels in all of the pre-treatment groups were nearly equivalent to the levels observed in the normal control group. However in the posttreatment group Ser (T): (9.9 ± 0.8 ng/mL) the level of T3 in the serum of mice was significantly increased but less than in pre-treatment groups.

### 3.9 Effect on IL-2 and IL-4

**Supplementary Fig. 2:**
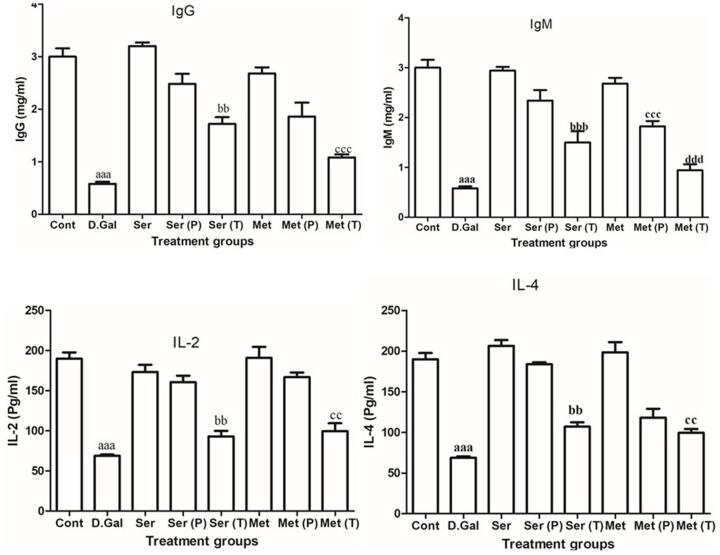
Effect of silk sericin on serum IL-2,IL-4, IgG and IgM

The administration of D-galactose (250 mg/kg b.w.) intraperitoneally for 60 sequential days resulted in the most significant decrease (68.8 ± 1.5 Pg/mL) in IL-2 when compared with a control group (189.8 ± 7.8 Pg /mL). Similarly, a noteworthy reduction (71.4 ± 4.2 Pg /mL) in IL-4 level was found when matched to the control group (185.8 ± 7.2 Pg /mL). Highest significant elevation in IL-2 levels (Ser (P): 160.4 ± 8.2 Pg /mL; Met (P): 171.2 ± 8. Pg /mL was perceived in prevention/Pre-treatment groups when compared with D-Gal group (68.8 ± 1.5 Pg /mL). However, IL-2 and IL-4 in the serum of mice were expressively increased to the highest extent in post-treatment groups, but this restoration was less than in all pre-treatment and individual groups as illustrated in Fig. 4. Maximum significant elevation in the level of IL-2 was found in a post-treatment group of (Ser (T): 92.8 ± 6.9 Pg /mL Met (T): 99.4 ± 9.9 Pg /mL with respect to D-Gal group (65.4 ± 1.7 Pg /mL). Similarly, a substantial upsurge in the level of IL-4 was found in a post-teratment group of (Ser (T): 107.0 ± 5.3 Pg /mL: Met (T): 97.4 ± 5.9 Pg /mL with respect to D-Gal group (71.4 ± 3.8 Pg /mL) as depicted in supplementary Fig. 2.

### 3.10 Effect on serum IgM and IgG

Supplementation of D-galactose (250 mg/kg body weight) via intraperitoneal for 60 days caused a noteworthy reduction (0.6 ± 0.0 mg/mL; 0.7 ± 0.1 mg/mL) in IgG and IgM respectively when matched to the control group (3.0 ± 0.2 mg/ mL). However, when sericin was given orally (150 mg/kg body weight) to mice, the highest significant upsurge was recorded as compared to D-Gal group. Administration of metformin also increased the level of serum IgG and IgM but is lower than sericin. On the other hand, the administration of sericin along with D-Gal the highest substantial reduction (p < 0.01) was observed when compared to D-Gal group. However, the highest significant attenuation was noticed in the level of serum immunoglobuLins in the post-treatment group of Sericin as revealed in supplementary Fig. 2.

### 3.11 Effect of sericin on length of telomere

The length of telomere of mice was assessed by qPCR. Real-time/qPCR data shows that the level of telomere length-related protein/ gene TERT significantly downregulated (9.4 ± 0.1) in D-Gal -treated group (250 mg/kg b.w.) with respect to normal saline treated group (23.0 ± 0.4). Whereas non-substantial fold change was observed in control and treatment groups of Actb gene/protein. The highest significant elevation (Ser: 24.74 ± 0.3, Met: 21.60 ± 0.5) in the telomere length was found in sericin, and metformin-treated group at a (150 mg/kg b.w.) dosage due to the upregulation of TERT. Pre-treatment/Co-administration of sericin and SNPs along with D-Gal caused a significant fold change (Ser (P): (17.93 ± 0.3); Met (P):(17.69 ± 0.2) in telomere length as compared to D-Gal /aged group (9.4 ± 0.1). These findings demonstrated that the telomere length in all pre-treatment groups was nearly equivalent to that of the untreated group. Whereas, in the post-treatment group, the level of TERT/telomere length mice was high and utmost substantial attenuated in (Ser (T): (14.99 ± 0.1); and Met (T): (12.49 ± 0.5) when compared to D-Gal group (9.4 ± 0.1) as shown in Fig. 5. In post-treatment groups, the expression of TERT increased but was lower than pre-treatment groups because mean values couLd not bring back to normal control levels.

### 3.12 Histology of liver

**Fig. 6:**
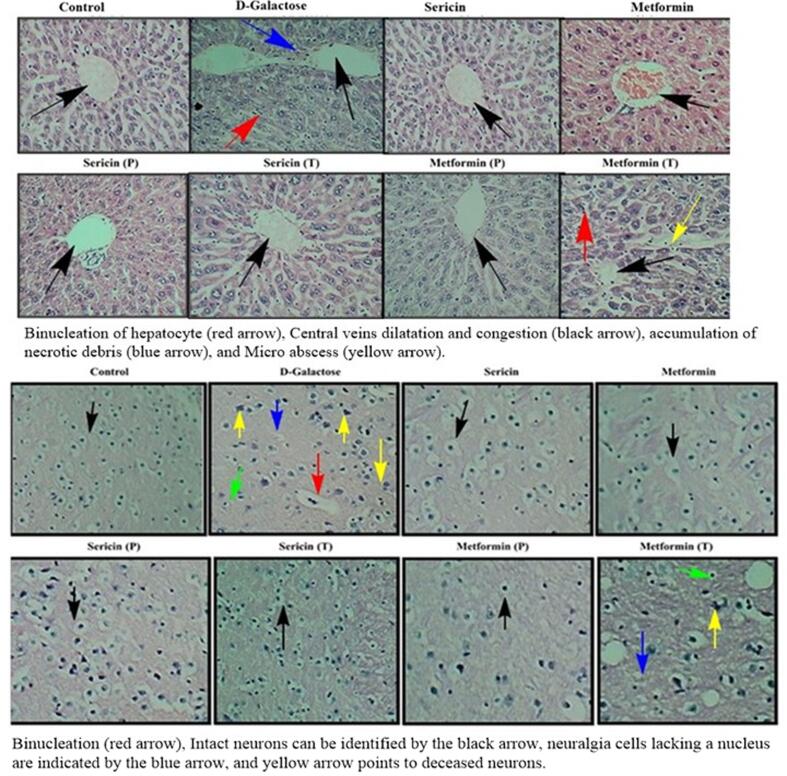
**Effect of sericin on histopathological changes induced by D-galactose in liver and brain of aged mice. Abbreviations and keys:** (H & E Staining; Magnification 10X). Binucleation of hepatocytes and neurons (red arrow), Central veins dilatation and congestion and intact neurons (black arrow), accumulation of necrotic debris in liver and neuralgia cells lacking a nucleus (blue arrow), and Micro abscess in liver and deceased neurons are indicated by yellow arrow. (For interpretation of the references to color in this figure legend, the reader is referred to the web version of this article.)

Histopathological features in the liver of aging-induced mice treated with silk proteins sericin and metformin were illustrated in Fig. 6. D-Gal induced aging group revealed morphological changes in swollen hepatocytes with infiltration of inflammatory cells, the liver such as microabscess involving a few hepatocytes, accumulation of necrotic debris, vacuolation, binucleation, Kupffer cell proliferation, extensive sinusoidal dilatation and congestion, enlarged intercellular space and ballooning degeneration of hepatocytes, central veins dilatation, and congestion. In contrast, the control group exhibited a liver with normal structure, including an intact central vein and organized hepatocytes with normal nuclei. Prevention with silk sericin significantly alleviated the liver changes in the group treated with D-Gal almost near to the untreated group. Post-treatment with silk sericin reversed the alterations caused by D-Gal to some extent but recovery in the post-treatment groups was less than in the prevention groups.

### 3.13 Histology of brain

The histological section of the brain showed that in the D-galactose group, neuronal loss was evident with a high level of inflammatory cells whereas in the control group, intact neurons were found. Furthermore, the group treated with D-Gal displayed a reduction in the number of pyramidal cells within the hippocampus region, accompanied by the presence of several atrophied and deteriorated cells. On the other hand, the control group demonstrated various well-arranged pyramidal cells and neuralgia cells dispersed within the matrix. Treatment with silk sericin as a preventive measure resulted in a compact film of pyramidal cells with vesicular nuclei in the D-Galactose treated group. However, the recovery was not as significant in the metformin-treated group. Furthermore, post-treatment with silk sericin helped mitigate the neuronal loss in the group treated with D-Gal, though this improvement was not as pronounced as in the prevention group. The brain section of the post-treatment group did not closely resemble that of the control group, unlike the prevention group. These outcomes suggest that silk sericin has the prospective to act as a neuroprotective agent against D-Gal-stimulated aging in animals as shown in Fig. 6.

## 4 Discussion

In cocoon formation, secondary metabolites such as flavonoids consumed in silkworms from the muLberry plants contribute to the composition of silk protein solution and the antioxidant features (Napavichayanun et al., 2017). Miguel and Álvarez-López (2020) stated that the total amount of flavonoid and polyphenolic content depends upon the methods of extraction such as autoclaving, acid degradation, alkali, and conventional. In previous studies, it was stated that in high thermal energy or autoclave method output of polyphenols was perceived higher whereas extraction of sericin through the urea method gave low revenue of flavonoid and polyphenolic contents. High phenolic content was found by breakage of peptide linkage among sericin and fibroin in the autoclave method. The high yield of flavonoids is the consequence of the breakage of glycosidic bonds as flavonoids are linked with silk proteins through glycosidic bonds (Napavichayanun et al., 2017, Anuduang et al., 2020). In the conducted research a high yield of total phenolic and total flavonoid content was perceived in silk sericin. Our results are supported by Miguel and Álvarez-López (2020) who quantified that degumming of silk sericin under high pressure and high temperature (autoclave) can yield maximum TFC from the silk proteins due to cleavage of the glycosidic bonds. Conversely, a low yield of flavonoids was found in a base-like extraction method such as sodium carbonate because it was incapable to disruption of the glycosidic bonds. In the present study, gallic acid (GA) was used as the standard to evaluate the TFC of silk proteins due to its purity and stability over two weeks as described by (Manesa et al., 2020). Moreover, the current study investigated the antioxidant activity of silk sericin at various concentrations (ranging from 0.5 to 2 mg/mL) using FRAP analysis and DPPH scavenging ability. Silk sericin is comprised of an impressive 78 % polar amino acids, specifically serine and threonine. These remarkable components contribute to its antioxidant properties due to hydroxyl groups, which possess the ability to chelate metallic elements such as Fe and Cu (Jena et al., 2018; Ghosh et al., 2020). Gurung et al. (2020) stated that in antioxidant characteristics of nutritional polyphenols, FRAP is an easy and well-known method. Maximum reduction ability of Fe3 + to Fe2 + in silk sericin was found when extracted through an autoclave. In the conducted research silk sericin showed the conversion of Fe3 + to Fe2 + at all concentrations of sericin but maximum reducing ability was found at the maximum meditation 2 mg/mL which showed that sericin consists of an enormous proportion of free reducing groups (Kumar and Mandal, 2017). In the current research, it was found that the D-Gal-induced aging group disclosed maximum symptoms of aging. Similar results were found in a previous study they also described that D-Gal induced aging mice. In our conducted experiment, sericin demonstrated a similar ability to scavenge free radicals compared to ascorbic acid, a standard antioxidant. Interestingly, as the concentration of sericin amplified ranging from 0.5 to 2 mg/mL, its antioxidant activity became even more pronounced. These findings are consistent with the research conducted by Manesa et al. (2020), which also observed that silk sericin exhibited a similar radical foraging action to Trolox. The presence of flavonoid and carotenoid pigments in argema mimosa sericin contributed to its high total phenolic content (TPC) and enhanced DPPH radical scavenging activity. Sericin is capable of scavenging the DPPH radical by donating a hydrogen atom, resulting in a color change from purple to yellowish (Manesa et al., 2020). According to Zhen et al. (2016), mice exhibited visible indicators of aging, including delayed response, reduced mobility, and dull and deteriorated fur. Moreover, a reduction in the body weight of the D-Gal group was found with respect to control group. However, the highest significant increase in the weight of mice was found in all prevention groups in comparison to D-Gal stimulated aged group.

In this research, the group treated with D-Gal showed the greatest decrease in antioxidant enzyme levels, including SOD, CAT, and GSHpx, compared to the untreated group. Whereas, oral supplementation of silk sericin attenuated the level of antioxidant enzymes. According to Silva et al. (2022), administering large amounts of sericin protein to rats resulted in significantly higher levels of antioxidant enzymes GSH-Px and SOD, compared to the aging group. Lee et al. (2020) have also found that the administration of ethanol considerably declines the activities of antioxidant-related enzymes. However, pretreatment with sericin protein restored or significantly upsurge the concentrations of antioxidant enzymes e.g., GSH-Px, SOD, and CAT.

Our outcomes are supported by Yuan et al. (2020) who also found that in the D-Gal model group, the serum levels of cortisol were considerably greater (P < 0.05) in comparison to the normal saline-treated group. Yuan et al. (2020) also discovered that the serum levels of cortisol were significantly higher (P < 0.05) in the D-Gal model group compared to the normal saline-treated group, supporting our findings. However, supplementation of ginseng stem-leaf saponinse (GSLS) to D-Gal exposed mice expressively reduced the level of cortisol as compared to the individual D-Gal group (Jeremy et al., 2017). Extensive reports have revealed that with age the levels of many hormones vary (Darst et al., 2019). According to Wu et al. (2020), the level of T3 in mice treated with a high-fat diet is reduced due to oxidative stress, but the administration of dietary methionine through oral dosage increases the level of T3. The same results have been found in our research as described by (Wu et al., 2020). Oxidative stress, nervousness, learning, remembrance, and intellectual capability are thoroughly connected to thyroid function (Villanueva et al., 2013). For regulating neural development and sustaining normal function of the central nervous system thyroid hormones are necessary (Ahmed and Mohammed, 2020).

The conducted research suggests that cytokines play an important role in regulating various physiological functions such as cell proliferation, differentiation, death, immune function, etc. However, in the aging host, the levels of cytokines also change and under certain abnormal conditions, excessive or aberrant production and action of cytokines can lead to various pathological conditions in cells and tissues as well as diseases in mammals (Asim et al., 2020). The current research indicates that the level of IL-2 and IL-4 decreased in D-Gal induced aging group and upregulation has been found in the silk sericin-treated group. According to Hu et al. (2022), Anwulignan reverses the inflamed aging process and regulates the level of cytokines in mice, thereby restoring immune function that is compromised during the aging process. In aging mice, Anwulignan increased the serum levels of IFN-γ, IL-2, and IL-4 while decreasing the levels of TNF-α and IL-6. In addition to controlling the growth and programmed cell death of activated T cells, IL-2 also stimulates B cells to produce immunoglobulins, which have anti-inflammatory properties. The immune system produces IL-4, which improves the immune response to antigens (Zhao et al., 2020). According to a study by Kumari et al. (2022), it was found that the levels of IL-2 and IL-4 were increased in the chick embryo (CE) and nutrient mixture (NM) group when compared with the D-galactose-induced aging mice model. The study also found that CE and NM reversed inflammatory cytokine levels in the serum. (Kumari et al., 2022).

The antibody system is a nonspecific immune factor that plays a crucial role in the body's immune response and immune adaptation (Li et al., 2020). Immunoglobulin (Ig) is a commonly used indicator of humoral immune status, and serum Ig includes IgG, IgM, IgA, IgD, and IgE. Among these, IgG and IgM are the main components of serum antibodies, as their content can reflect the immune response capacity and humoral immunity of the body (Ladomenou and Gaspar, 2016). In the current study, the serum levels of IgG and IgM were examined to reflect the body's humoral immune level and immune response capacity, which also fluctuate as a person ages (Li et al., 2020). The results of the study showed that, in comparison to a control group, the D-galactose-induced aging group had significantly fewer immunoglobulins. According to Li et al. (2020), this reduction may lower the humoral immune response level. Gao et al. (2018) provided support for these findings, reporting that Anwulignan administration increased these immunoglobulins and reversed the effects of D-gal. This suggests that Anwulignan has a regulatory role in the humoral immune response in aging mice. Furthermore, it has also been found that the level of immunoglobulins (IgG and IgM) decreased in the serum of D-Galactose induced aging group of mice as compared to the normal group. Whereas administration of chitosan via gavage elevated the level of immunoglobulins in a dose-dependent manner (Kong et al., 2019).

In the conducted research anti-aging effect of sericin was assessed by observing the expressions of TERT /telomere length through qPCR. Real-time data showed that the level of telomere length-related protein/gene TERT significantly downregulated in D-Gal -treated group (250 mg/kg b.w.) with respect to normal saline treated group. Whereas the highest significant elevation in telomere length was observed in sericin. The increase in elevation could be attributed to the various beneficial effects of silk proteins, such as their antioxidant activity, ability to prevent blood clotting, reduce inflammation, fight against tumors, and promote anti-aging and anti-wrinkle effects (Jo et al., 2020, Jo et al., 2021). Our results are supported by Chen et al. (2017) who found that the D-Galinduced aging mice model showed significantly (P < 0.05) shorter hepatic telomere length than the control group. Whereas supplementation of polyunsaturated fatty acids (PUFA) in the aging mice model significantly prevents the excessive attrition of telomere (Kiecolt-Glaser et al., 2013). Similarly, administration of high, medium, and low doses of fish oil in the aging mice model significantly increased the hepatic telomere length within a range of 13–27 % (Chen et al., 2017). Substantially, higher telomerase activities in that aging model group have been found with shorter telomeres compared with the untreated group. It has been stated that limiting telomerase levels is required for the homeostasis of telomere length (Srinivas et al., 2020). It has been also found that a high dose of fish oil suppresses telomerase activity whereas, a moderate dosage showed a telomere-protective effect. TERT is a catalytic subunit of telomerase that can be regulated both epigenetically and transcriptionally. In the aged rats, no significant change (P > 0.05) in the TERT mRNA expression has been found. However, administration of both docosahexaenoic acid (DHA) and fish oil (FO2) at small and modest dosages significantly upregulated the expression of TERT protein within the ranges of 26 %-36 %, and 25 %-45 % correspondingly (Dhillon et al., 2021). In previous research, it has also been found that administration of DLBS1649/Riboflavin at a dosage of 50 μg/ mL upregulated the TERT protein level (28 %) in all treatment groups with respect to the aged group and this upregulation occurred due to maintained activity of telomerase (Fitrawan et al., 2018). Cell senescence is categorized through the shortening of telomeres. Telomerase enzyme maintains telomere length and prevents aging. Karsono et al. (2021) stated that telomere shortening has also been found in DLBS1649/riboflavin-treated cells but in a slower manner which suggests that treated cells still went through aging. These consequences designated that DLBS1649 has an antiaging effect and increases the life span without stimulating cancerous characters in cells as observed in our study. In previous studies, it has also been conveyed that the hepatic system of the D-Gal-induced aging group had partial necrosis, swelling, inflammatory cell infiltration, and edema in hepatocytes at 120 mg/kg b.w and 60 mg/kg b.w (D-Gal) in contrast to control group. Our outcomes agree with earlier studies of Kong et al. (2018) who described that D-Gal caused hepatic and renal structure destruction such as necrosis of hepatocytes, and atrophy of the renal glomerulus whereas; administration of vitamin E and chitosan alleviated these changes closely to that control group. In the present study, the D-Gal group showed a decrease in the number of pyramidal cells and distortion of neurons with respect to the normal saline treated group. Whereas in prevention groups Ser (P) and Met (P) respectively showed abundant intact neurons similar to that control group but recovery was found less in metformin treated group than sericin treated group. However, in all post-treatment groups, it was found that some neurons were intact and some were distorted to some extent but this distortion was less than in the aging group. Chiroma et al. (2018) found that intraperitoneal injection of D-Gal in mice caused deterioration of neuroglial cells, impairments in their hypothalamus, and intellectual injuries. Our findings align with the results recounted by Wang et al. (2021c) who validated that the control group exhibited a condensed layer of neurons with clear nucleoli as compared to the aging group. Whereas, co-administration with WIN, caused a lower structural aberration and significantly reduced the number of pyknotic neurons, than that of D-Gal groups. As a result, the findings of this study demonstrated that WIN has the ability to promote neurogenesis in neurons of aged rats and can potentially protect and maintain their normal cytoarchitecture (Mahdi et al., 2021).

## 5 Conclusions and future perspectives

Oxidative stress, resulting from an excessive production of reactive oxygen species (ROS), primarily influences the aging process. This can also weaken the immune system, increasing the likelihood of age-related diseases. To combat this, there is interest in exploring different substances with anti-inflammatory and antioxidant properties that could potentially improve anti-aging treatments. Natural compounds like sericin, found in silk, have been studied for their antioxidant properties and potential benefits in slowing down or treating aging. Our research demonstrates that sericin from silk has various applications, including as an antioxidant, anti-aging agent, antibacterial substance, and ROS scavenger. It is also a more cost-effective option for delaying and preventing aging and age-related diseases. Silk proteins have been found to possess antioxidant and antiaging properties and these properties are attributed to their ability to decrease oxidative stress and enhance the activity of antioxidant defense enzymes, such as CAT, SOD, and GSH as discussed. Additional research is necessary to enhance the demand for silk products to benefit socioeconomic welfare by using genetically modified silkworms. Moreover, in-depth in vivo research and clinical trials are necessary to explore the potential applications of silk products in the cosmetics and pharmaceutical industries. It is also required to explore the bioactive properties so that it can be safely used as a functional nutrition constituent. Therefore, it is recommended to intake food enriched with flavonoids and antioxidants to prevent or slow down the process of aging.

## Declaration of competing interest

The authors declare that they have no known competing financial interests or personal relationships that could have appeared to influence the work reported in this paper.
